# St John's Wort (*Hypericum perforatum*) Oil on Immune Response, Growth Performance and Bacterial Resistance Against *Lactococcus garvieae* Pathogen in Rainbow Trout (*Oncorhynchus mykiss*)

**DOI:** 10.1111/jfd.70157

**Published:** 2026-03-18

**Authors:** Soner Bilen, Mustafa Karga, Osman Nezih Kenanoğlu, Ertuğrul Terzi

**Affiliations:** ^1^ Ihsangazi Vocational School Kastamonu Universitesi Kastamonu Turkiye; ^2^ Inebolu Vocational School Kastamonu Universitesi Kastamonu Turkiye; ^3^ Food Engineering, Faculty of Engineering and Architecture Kastamonu Üniversitesi Kastamonu Turkiye; ^4^ Devrekani TOBB Vocational School Kastamonu Üniversitesi Kastamonu Turkiye

**Keywords:** fish immunology, growth performance, *Hypericum perforatum*, immunostimulant, *Lactococcus garvieae*

## Abstract

In this study, we investigated the immunomodulatory effects of St. John's Wort ( *Hypericum perforatum* ) oil on the growth performance, immune responses and resistance against *Lactococcus garvieae* in rainbow trout ( *Oncorhynchus mykiss* ). The plant oil was incorporated into feeds at concentrations of 1, 3, and 5 g kg^−**1**^, designated as K1, K3, and K5, using a spray‐coating method. A total of 12 tanks (100 L each) were used, with three replicates per group and 40 fish (10–11 g) stocked per tank. Trout fed with St. John's Wort oil exhibited significant improvements in immune parameters, particularly at the transcriptional level. Notably, after 40 and 60 days of feeding, the K3 group showed significant upregulation of immune‐related genes, including *IL‐1β, IL‐8, IFN‐1, TNF‐α, COX‐2, TGF‐β*, and *MHC‐II* in kidney and intestinal tissues. Following experimental challenge, survival rates reached 60% in K5, 50% in K3, and 35% in K1, compared with 30% in the control group. After 60 days, final body weights were 32.60 ± 0.34 g (K1), 34.83 ± 0.29 g (K3), 30.80 ± 1.46 g (K5), and 26.42 ± 0.36 g (control). The K3 group showed significantly higher final weight compared with all other groups (*p* < 0.05), whereas no significant differences were detected among the remaining treatments. These findings indicate that dietary inclusion of *H. perforatum* oil at 3 g kg^−1^ enhances immune gene expression and disease resistance without compromising growth performance, suggesting its potential as an immunonutritional strategy in trout aquaculture.

## 1 Introduction

While the need for nutritional protein is increasing in the world, aquaculture production, which is the fastest growing animal production sector, has become an indispensable part of the food supply today (OECD‐FAO 2016). With the decline in capture fisheries, aquaculture production has expanded rapidly and has become a major strategy to meet the growing demand for high‐quality protein sources. The Food and Agriculture Organization of the United Nations (FAO) recently announced in its 2022 report that aquaculture is the fastest growing sector in the agricultural sector. Rainbow trout ( *Oncorhynchus mykiss* ) farming constitutes an important industry among cultured fish species in many countries (FAO 2022). It is also one of the most produced cultured fish species in Turkiye. However, the rapid intensification of aquaculture practices has increased disease pressure, particularly under high stocking densities and intensive rearing conditions, posing significant challenges for sustainable production.

Disease‐associated mortality remains a major constraint in aquaculture production. Although there are methods such as vaccination and antibiotic use to prevent diseases, their application is often limited by economic, logistical, and environmental concerns. Vaccination strategies can be costly and labor‐intensive, particularly in small‐sized fish. However, the excessive and uncontrolled use of antibiotics has contributed to the emergence of antimicrobial resistance, which represents a critical challenge in aquaculture. In addition, antibiotic residues may accumulate in aquatic environments, promoting resistant bacterial strains and posing ecological risks. As an alternative strategy, dietary immunostimulants have gained increasing attention as sustainable approaches to enhance host defence mechanisms. In recent years, plant‐derived bioactive compounds have been extensively investigated for their immunomodulatory properties in fish. Several medicinal plants have been shown to enhance disease resistance and modulate innate immune responses in teleost species (Özçelik et al. 2020). These phytogenic compounds may also act as functional feed additives, supporting growth performance while simultaneously strengthening immune competence in commercial aquaculture systems (Bilen et al. 2016; Fılogh et al. 2023).

St. John's Wort ( *Hypericum perforatum* ) is a medicinal plant widely distributed in North Africa, Europe, and West Asia, and naturally abundant in Turkiye, with reported antibacterial and antioxidant properties (Farzollahi et al. 2019). *H. perforatum* has been evaluated as a dietary additive in fish (Çilingir et al. 2017) and as a potential immunostimulant (Farzollahi et al. 2019), with evidence suggesting its capacity to modulate immune responses and enhance disease resistance in aquaculture species.

*Lactococcus garvieae* is the bacterium that causes lactococcosis, a hyperacute, hemorrhagic septicemia in fish. In addition to its impact on aquaculture, *L. garvieae* is recognised as a zoonotic pathogen capable of causing infections in humans. Lactococcosis leads to significant economic losses in aquaculture, affecting numerous freshwater and marine species of commercial importance (Meyburgh et al. 2017). In rainbow trout, *L. garvieae* infection is characterised by systemic involvement and rapid mortality (Eldar and Ghittino 1999; Chang et al. 2002). This not only causes the deaths of intensively farmed rainbow trout, but also directly causes intense economic losses.

In the present study, we aimed to evaluate the protective and immunomodulatory effects of St. John's Wort ( *Hypericum perforatum* ) oil against *Lactococcus garvieae* , a major bacterial pathogen in rainbow trout. The potential of *H. perforatum* oil as a dietary immunostimulant and therapeutic feed additive capable of enhancing immune responses over a 60‐day period without adversely affecting growth performance was evaluated.

The present study was designed to determine the optimal immunostimulatory and protective dose of *H. perforatum* oil in rainbow trout, to evaluate the appropriate duration of dietary application, and to assess its potential as a natural feed additive capable of enhancing immune competence and growth performance without adverse environmental impacts.

In addition, the study aimed to explore whether this phytogenic strategy could serve as a sustainable alternative to conventional chemotherapeutic, antibiotic, and vaccination‐based approaches in aquaculture. In particular, the comparative evaluation of different dietary inclusion levels demonstrated that the K3 dose induced significant upregulation of immune‐related genes, suggesting a coordinated response at both the physiological and molecular levels. Furthermore, the findings support a dose‐optimisation strategy based on experimental evidence rather than empirical supplementation, highlighting the potential of this phytogenic additive as part of an immunonutritional approach in aquaculture. In this context, the study contributes to the growing body of literature on dose‐dependent phytogenic immunostimulation in fish, providing evidence for a dose‐sensitive and environmentally compatible feed additive strategy.

## 2 Materials and Methods

### 2.1 Preparation of Feeds

The feeds used in the experiment were provided free of charge by a commercial enterprise. Plant oil was incorporated into the feeds at inclusion levels of 1, 3, and 5 g kg^−1^ using a spray‐coating method and subsequently vacuum‐packaged. To ensure uniform distribution, the oil was thoroughly mixed before vacuum packaging. To minimise lipid oxidation, the diets were vacuum‐packaged and stored at −20°C in the dark until use. The proximate composition of the basal diet is presented in Table 1.

**TABLE 1:** The composition of the fish feed used in the study.

The nutrient composition of the feed (%)	
Crude Protein	44
Crude Oil	18
Crude Cellulose	2
Raw Ash	10
Phosphorus	1.5
Calcium	2
Moisture	11
Omega 3	1
Omega 6	1.5
EPA + DHA	5
Other	4

### 2.2 Experimental Fish and Rearing Conditions

The feeding trial was conducted at the Aquatoxicology Unit of the Faculty of Fisheries, Kastamonu University. Prior to fish stocking, the aquaria were thoroughly cleaned, rinsed with freshwater, allowed to dry, and the system was filled and checked for proper functioning. A total of 650 rainbow trout (10–11 g) were obtained from a commercial trout farm to compensate for potential losses during transportation and acclimation. Fish were acclimated to laboratory conditions for 15 days. Following acclimation, 480 clinically healthy fish were randomly selected and distributed into 12 closed‐circuit aquaria (100 L each), with 40 fish per tank. The experimental design consisted of three dietary treatment groups and one control group, each with three replicates. Each tank was individually aerated throughout the experimental period, and approximately 25% of the total water volume was renewed daily with fresh water. Water temperature was maintained at 14°C–16°C using a Teco TK 5 K chiller–heater system. pH was monitored daily and ranged between 6.5 and 7.0, while dissolved oxygen levels were maintained between 8.6 and 10.2 mg L^−1^. Fish were hand‐fed three times daily to apparent satiation.

### 2.3 St. John's Wort Oil Characterisation by GC–MS

St. John's Wort ( *Hypericum perforatum* ) oil used in the study was commercially obtained, and its chemical composition was analysed using gas chromatography–mass spectrometry (GC–MS) at the Kastamonu University Central Laboratory. The identified compounds and their relative abundance are presented in Table 2.

**TABLE 2:** St. John's wort ( *Hypericum perforatum* ) GC–MS analysis report.

No	Molecule	Percentage
1	Oleic Acid	51.17
2	9‐Octadecenoic acid (Z)‐, 2,3‐dihydroxypropyl ester	14.57
3	Palmitic acid	4.76
4	9‐Octadecenoic acid, 1,2,3‐propanetriyl ester (E,E,E)—	4.71
5	Hexadecanoıc Acıd, Ethyl Ester	4.10
6	Palmitoyl chloride	3.93
7	Oleoyl chloride	3.11
8	Hexadecanoic acid, 1‐(hydroxymethyl)‐1,2‐ethanediyl ester	3.01
9	xadecanoic acid, 1‐[[[(2‐aminoethoxy)hydroxyphosphinyl]oxy]methyl]‐1,2‐ethanediy	2.17
10	Hexadecanoic acid, 2‐hydroxy‐1‐(hydroxymethyl)ethyl ester	2.07
11	Oleic acid, trimethylsilyl ester (CAS)	1.31
12	Oleoyl chloride	0.98
13	Octadecanoic acid, methyl ester (CAS)	0.71
14	9‐Octadecenoic acid, methyl ester, (E)—	0.53
15	Octadecanoic acid, 2,3‐dihydroxypropyl ester	0.25
16	Hexadecanoic acid, methyl ester	0.24
17	4,8,12,16‐Tetramethylheptadecan‐4‐olide	0.24
18	cis‐11‐Eicosenoic acid, methyl ester	0.20
19	l‐(+)‐Ascorbic acid 2,6‐dihexadecanoate	0.20
20	Octadecanoic acid, 2,3‐dihydroxypropyl ester	0.14
21	Stearate <ethyl‐>	0.14
22	9‐Octadecenoic acid, 1,2,3‐propanetriyl ester (E,E,E)—	0.14
23	Dodecanoic acid, 2‐hexen‐1‐yl ester	0.13
24	9,12‐Octadecadienoic acid (Z,Z)‐, methyl ester (CAS)	0.10
25	11‐Octadecenoic acid, methyl ester	0.10
26	Other	1.05

### 2.4 Taking Blood and Tissue Samples From Fish

Blood and tissue sampling was performed on days 20, 40, and 60 of the feeding trial. Fish selected for sampling were anaesthetised with 2‐phenoxyethanol (3 mL L^−1^) prior to handling (Mylonas et al. 1998). Blood samples were collected from the caudal vein using sterile 1 mL syringes. The puncture site was disinfected with 70% ethanol before sampling. Blood samples were transferred into K_3_EDTA tubes for haematological analyses and serum separator tubes for immunological assays. Serum was obtained by centrifugation at 4°C and stored at −20°C until analysis. For gene expression and immunological analyses, kidney and intestine tissues were aseptically excised using sterile dissection instruments. Tissue samples were immediately processed or stored under appropriate conditions for subsequent analyses.

### 2.5 Innate Immune Parameters

#### 2.5.1 Respiratory Burst Activity

Respiratory burst activity was determined using the nitroblue tetrazolium (NBT) reduction assay according to Siwicki et al. (1994). Briefly, 0.1 mL of whole blood was mixed with 0.1 mL of 0.2% NBT solution and incubated at room temperature for 30 min. After incubation, 50 μL of the mixture was transferred to a glass tube, and 1.0 mL of N,N‐dimethylformamide was added to terminate the reaction. Samples were centrifuged at 4000 × g for 5 min, and the supernatant was collected. Absorbance was measured at 540 nm against N,N‐dimethylformamide as blank. Results were expressed as optical density (OD540).

#### 2.5.2 Lysozyme Activity

Lysozyme activity was determined using a turbidimetric assay according to Sankaran and Gurnani (1972). A 0.02% suspension of *Micrococcus lysodeikticus* prepared in phosphate buffer (pH 6.2) was used as substrate. Serum samples were added to the substrate solution and incubated at 25°C, and the decrease in absorbance was monitored spectrophotometrically. Lyophilized chicken egg white lysozyme was used as standard for calibration. Results were expressed as mg egg white lysozyme equivalents mL^−1^.

#### 2.5.3 Myeloperoxidase Activity

Myeloperoxidase (MPO) activity was measured with slight modifications of the method described by Seeley et al. (1990) and Sankaran and Gurnani (1972). Briefly, 60 μL of serum was diluted with 540 μL of Hanks' balanced salt solution (HBSS). Subsequently, a substrate solution containing 3,3′, 5,5′‐tetramethylbenzidine dihydrochloride (TMB) and hydrogen peroxide was added, and the reaction was stopped after 2 min by adding 220 μL of sulfuric acid. Absorbance was measured at 450 nm, and MPO activity was expressed as optical density (OD450).

### 2.6 Analysis of Cytokine Gene Expression

#### 2.6.1 RNA Isolation From Tissue Samples

On days 20, 40, and 60 of the feeding trial, three fish per group were randomly sampled. Fish were anaesthetised with 2‐phenoxyethanol prior to dissection, and head kidney and intestine tissues were aseptically excised. Tissue samples were immediately transferred into Invitrogen RNAlater solution and subsequently stored at −80°C until RNA extraction. Total RNA was isolated from tissue samples using a commercial RNA isolation kit (FavorPrep FATRS 100) according to the manufacturer's instructions. RNA concentration and purity were determined spectrophotometrically by measuring absorbance at 260 and 280 nm, and the A260/A280 ratio was used as an indicator of RNA quality.

#### 2.6.2 Complementary DNA (cDNA) Synthesis

Complementary DNA (cDNA) was synthesized using the High‐Capacity cDNA Reverse Transcription Kit (Applied Biosystems, USA) according to the manufacturer's instructions. Reverse transcription reactions were prepared in a total volume of 20 μL, containing 2.0 μL of 10× RT buffer, 0.8 μL of 25× dNTP mix (100 mM), 2.0 μL of 10× random primers, 1.0 μL of MultiScribe reverse transcriptase (50 U μL^−1^), and 15 ng of total RNA. Nuclease‐free water was added to complete the final volume. The thermal cycling conditions were as follows: 25°C for 10 min, 37°C for 120 min, and 85°C for 5 min, followed by cooling at 4°C. The quality and concentration of the synthesized cDNA were assessed by agarose gel electrophoresis and spectrophotometric analysis. The synthesized cDNA was stored at −20°C until RT‐qPCR analysis.

#### 2.6.3 Determination of Cytokine Gene Expression by RT‐qPCR

Gene expression levels of *IL‐1β, IL‐8, IFN‐1, TGF‐β, TNF‐α, COX‐2*, and *MHC‐II* were quantified by reverse transcription quantitative PCR (RT‐qPCR) using gene‐specific primers and SYBR Green I chemistry on a CFX96 Touch Real‐Time PCR Detection System (Bio‐Rad, USA). β‐actin was used as the reference gene. Primers previously validated for rainbow trout were used, and qPCR reactions were prepared using a 2× SYBR Green Master Mix according to the manufacturer's instructions. Each reaction was performed in triplicate, and no‐template controls (NTCs) were included in each run. Amplification was carried out under the following conditions: initial denaturation at 95°C for 3 min, followed by 40 cycles of 95°C for 15 s and primer‐specific annealing/extension at 60°C for 30 s. Melting curve analysis was performed from 65°C to 95°C with 0.5°C increments to confirm amplification specificity. Cycle threshold (Ct) values were obtained using the CFX Maestro software (Bio‐Rad). Relative gene expression levels were calculated using the 2^−ΔΔCt^ method. Rainbow trout reverse and forward primers are given in Table 3.

**TABLE 3:** Rainbow trout reverse and forward primers.

Gene	Primers (Forward and Reverse)	References
*β‐Aktin*	F5′ ATGGAAGGTGAAATCGCC 3′	Sigh et al. (2004)
	R5′ TGCCAGATCTTCTCCATG 3′	
*IL‐1β*	F5′ ACCGAGTTCAAGGACAAGGA 3′	Awad et al. (2011)
	R5′ CATTCATCAGGACCCAGCAC 3′	
*IL‐8*	F5′ CACAGACAGAGAAGGAAGGAAAG 3′	Awad et al. (2011)
	R5′ TGCTCATCTTGGGGTTACAGA 3′	
*IFN‐1*	F5′ AGAATGCCCCAGTCCTTTTCC 3′	Ooi et al. (2008)
	R5′ GACTTTGTCCTCAAACTCAGCATCA 3′	
*TGF‐ β*	F5′ AGATAAATCGGAGAGTTGCTGTG 3′	Awad et al. (2011)
	R5′ CCTGCTCCACCTTGTGTTGT 3′	
*TNF‐α*	F5′ CAAGAGTTTGAACCTTGTTCAA 3′	Panigrahi et al. (2007)
	R5′ GCTGCTGCCGCACATAGAC 3′	
*COX‐2*	F5′ GGGCTTTGACATCCTCAACA 3′	Chettri et al. (2011)
	R5′ CATCGGACAAGAACCCTTGA 3′	
*MHC2*	F5′ ATGTCGATGCCAATTGCCTTCTA3′	Sigh et al. (2004)
	R5′ TGTCTTGTCCAGTATGGCGCT 3′	

### 2.7 Growth Performance

Growth performance was evaluated on days 20, 40, and 60 of the feeding trial. Feed conversion ratio (FCR), specific growth rate (SGR), and weight gain (WG) were calculated using the following formulas:

$$\mathrm{FCR}=\text{Feed intake}\;\left(g\right)/\text{Weight gain}\;\left(g\right)$$

$$\begin{array}{cc} \mathrm{SGR}\;\left(\%{\mathrm{day}}^{-1}\right)=\; & [\left(\ln\;\text{final body weight}-\ln\;\text{initial body weight}\right)/\\ & \text{experimental period}\;\left(\text{days}\right)]\times 100 \end{array}$$

$$\mathrm{WG}\;\left(\%\right)=\left[\left(\text{final weight}-\text{initial weight}\right)/\text{initial weight}\right]\times 100$$

### 2.8 Bacterial Challenge Test

A bacterial challenge test was conducted to evaluate disease resistance against *Lactococcus garvieae* . Prior to the challenge, the median lethal dose (LD_50_) was determined in rainbow trout. At the end of the 60‐day feeding trial, fish were intraperitoneally injected with 0.1 mL of *L. garvieae* suspension at a concentration of 1 × 10^8^ CFU mL^−1^, prepared in sterile phosphate‐buffered saline (PBS), following the method described by Bilen et al. (2016). Fish were monitored daily for 14 days, and cumulative survival rates were calculated for each group.

Survival rate (SR, %) was calculated as:

$$\mathrm{SR}\;\left(\%\right)=\left(\text{number of surviving fish}/\text{number of challenged fish}\right)\times 100.$$

### 2.9 Statistical Analysis

All data are presented as mean ± standard error (SE). Statistical analyses were performed using SPSS software (version 23.0; IBM Corp., USA). Differences among experimental groups were evaluated by one‐way analysis of variance (ANOVA). When significant differences were detected, Tukey's multiple comparison test was applied to compare group means. Statistical significance was accepted at *p* < 0.05.

## 3 Results

### 3.1 Growth Performance

At the end of the 60‐day feeding trial, final body weight differed among experimental groups. The highest final weight was recorded in the K3 group (34.83 ± 0.29 g), followed by K1 (32.60 ± 0.34 g) and K5 (30.80 ± 1.46 g), while the lowest value was observed in the control group (26.42 ± 0.36 g). The final body weight of the K3 group was significantly higher than that of the other groups (*p* < 0.05), whereas no significant differences were detected among the remaining groups (*p* > 0.05). Feed conversion ratio (FCR) was lowest in the K3 group (1.45 ± 0.01); however, this difference was not statistically significant (*p* > 0.05). Specific growth rate (SGR) was significantly higher in the K3 group compared to the other groups (*p* < 0.05), while no significant differences were detected among the remaining treatments (*p* > 0.05). Similarly, weight gain was significantly higher in the K3 group compared to all other groups (*p* < 0.05). Detailed growth performance data are presented in Table 4.

**TABLE 4:** Growth performance data obtained at the end of the study.

	IW (g)	FW (g)	FCR	SGR	WG (%)
Control	10.11 ± 0.29^a^	26.42 ± 0.36^b^	1.62 ± 0.01^a^	2.14 ± 0.03^b^	161.42 ± 3.91^b^
K1	10.35 ± 0.15^a^	32.60 ± 0.34^b^	1.47 ± 0.06^a^	2.54 ± 0.07^b^	214.08 ± 9.58^b^
K3	10.79 ± 0.08^a^	34.83 ± 0.29^a^	1.45 ± 0.01^a^	2.60 ± 0.01^a^	222.76 ± 0.24^a^
K5	10.35 ± 0.08^a^	30.80 ± 1.46^b^	1.51 ± 0.15^a^	2.41 ± 0.13^b^	197.68 ± 16.86^b^

Abbreviations: FCR, feed conversion ratio; FW, final weight; IW, initial weight; SGR, specific growth rate; WG, weight gain. Different superscripts indicate significant differences among groups (*p* < 0.05). All datas analyzed by one‐way ANOVA and the TUKEY test and are presented as mean ± standard error (SE) (*n* = 9).

### 3.2 Changes in Immune Responses

#### 3.2.1 Respiratory Burst Activity (NBT Assay)

**FIGURE 1:**
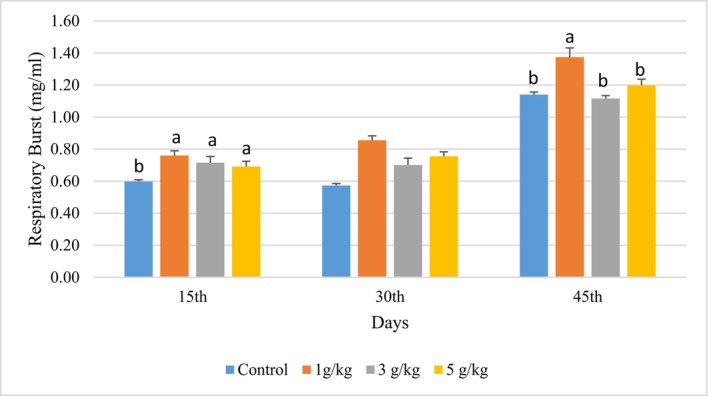
Respiratory burst activity (NBT assay, OD540) in rainbow trout fed diets supplemented with *Hypericum perforatum* oil for 60 days.

Respiratory burst activity was evaluated using the NBT reduction assay on days 20, 40, and 60 of the feeding trial (Figure 1). On day 20, NBT values were 0.76 ± 0.03 (K1), 0.72 ± 0.04 (K3), and 0.69 ± 0.03 (K5), and no significant differences were detected among these groups (*p* > 0.05). In contrast, the control group exhibited a lower NBT value (0.60 ± 0.01), which was significantly different from the supplemented groups (*p* < 0.05). On day 40, respiratory burst activity did not differ significantly among the experimental groups (*p* > 0.05). On day 60, the highest NBT value was observed in the K1 group (1.37 ± 0.06), which was significantly higher than the other groups (*p* < 0.05).

#### 3.2.2 Lysozyme Activity

**FIGURE 2:**
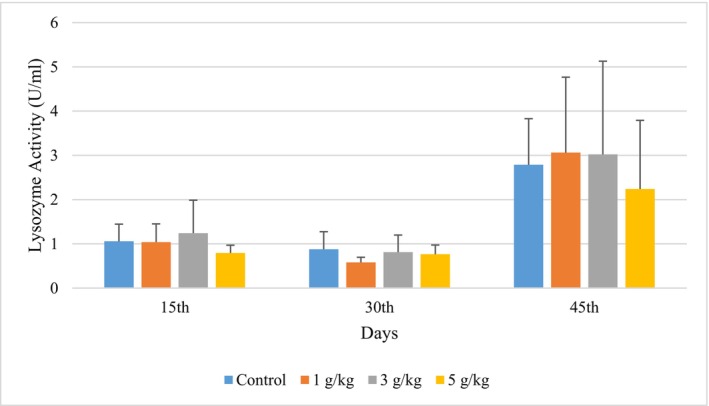
Lysozyme activity (U mL^−1^) in rainbow trout fed diets supplemented with *Hypericum perforatum* oil for 60 days. Different letters indicate significant differences among groups (*p* < 0.05).

Changes in the lysosome activity of fish on the 20th, 40th, and 60th days of the experiment are given in Figure 2.

On day 20, lysozyme activity was 1.0566 ± 0.38 (Control), 1.0386 ± 0.41 (K1), 1.2422 ± 0.74 (K3), and 0.8030 ± 0.18 (K5). Although the K3 group exhibited the highest value, no significant differences were detected among groups (*p* > 0.05). On day 40, lysozyme activity was 0.8777 ± 0.39 (Control), 0.5797 ± 0.12 (K1), 0.8116 ± 0.39 (K3), and 0.7647 ± 0.20 (K5), with no statistically significant differences observed (*p* > 0.05). Similarly, on day 60, lysozyme activity did not differ significantly among treatments (*p* > 0.05), despite numerical variation among groups.

#### 3.2.3 Myeloperoxidase Activity

**FIGURE 3:**
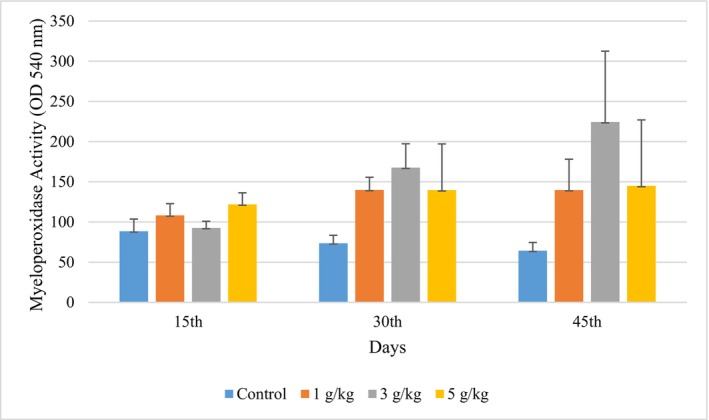
Myeloperoxidase activity (OD450) in rainbow trout fed diets supplemented with *Hypericum perforatum* oil for 60 days. Different letters indicate significant differences among groups (*p* < 0.05).

Myeloperoxidase activity results obtained at the end of the study are given in Figure 3.

On day 20, MPO activity showed numerical increases in the K1 and K5 groups compared to the control and K3 groups; however, no statistically significant differences were detected among treatments (*p* > 0.05). On day 40, MPO activity was numerically higher in K3 (167.606 ± 29.70), K1 (139.94 ± 15.66), and K5 (139.60 ± 57.54) compared to the control group (73.55 ± 9.92), but these differences were not statistically significant (*p* > 0.05). Similarly, on day 60, although higher MPO values were observed in K3 (167.606 ± 29.70), K5 (144.89 ± 82.11), and K1 (139.81 ± 38.25) compared to the control (64.19 ± 10.30), no significant differences were found among groups (*p* > 0.05).

#### 3.2.4 Gene Expressions

##### 3.2.4.1 IL‐1β

**FIGURE 4:**
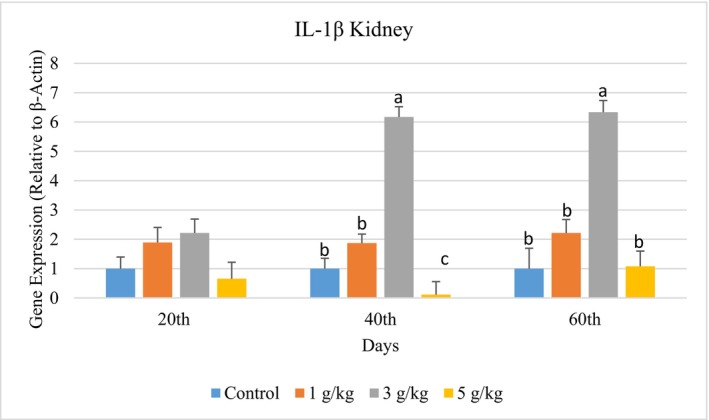
Relative IL‐1β gene expression in kidney tissue of rainbow trout fed diets supplemented with *Hypericum perforatum* oil for 60 days. Values are expressed as fold change (2^−^ΔΔCt). Different letters indicate significant differences (*p* < 0.05).

**FIGURE 5:**
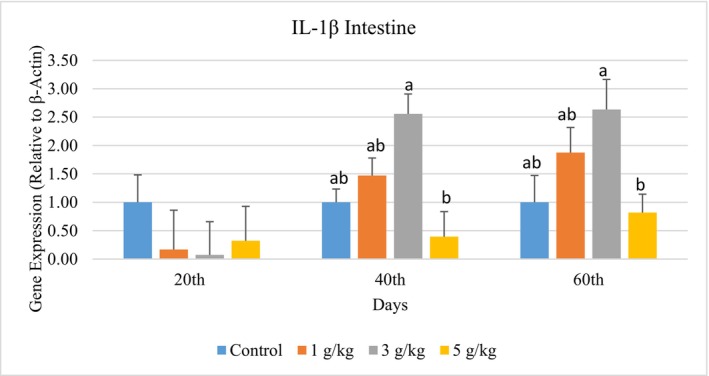
Relative IL‐1β gene expression in intestine tissue of rainbow trout fed diets supplemented with *Hypericum perforatum* oil for 60 days. Values are expressed as fold change (2^−^ΔΔCt). Different letters indicate significant differences (*p* < 0.05).

Relative *IL‐1β* gene expression levels in kidney and intestine tissues are presented in Figures 4 and 5.

In kidney tissue, *IL‐1β* expression remained unchanged among groups at day 20 (*p* > 0.05). A significant upregulation was observed in the K3 group at day 40 (*p* < 0.05), whereas K1 did not differ from the control. This pattern persisted at day 60, with the K3 group maintaining significantly higher expression levels compared to the other treatments (*p* < 0.05), while no significant differences were detected among the control, K1, and K5 groups.

In intestinal tissue, *IL‐1β* expression did not differ significantly among treatments at day 20 (*p* > 0.05). A similar trend was observed at later sampling points; however, the K3 group exhibited a significant increase relative to the control (*p* < 0.05), whereas the remaining groups did not show statistically significant changes.

##### 3.2.4.2 IL‐8

**FIGURE 6:**
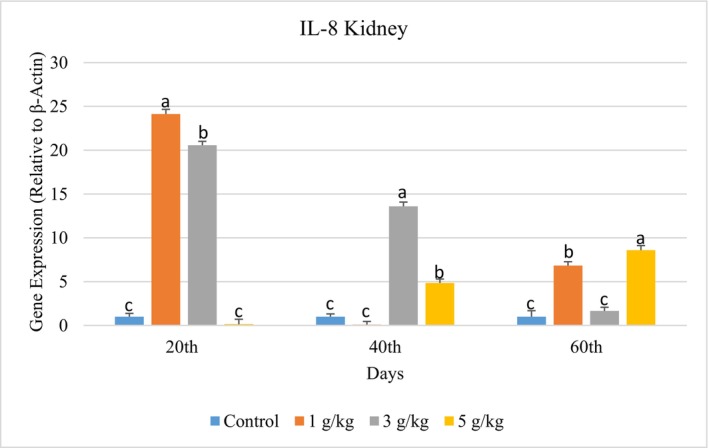
Relative IL‐8 gene expression in kidney tissue of rainbow trout fed diets supplemented with *Hypericum perforatum* oil for 60 days. Expression levels are presented as fold change (2^−^ΔΔCt). Different letters indicate significant differences (*p* < 0.05).

In kidney tissue, *IL‐8* expression exhibited a time‐ and dose‐dependent modulation. At the first sampling point, expression levels were significantly elevated in the K1 group compared to the control and K5 groups (*p* < 0.05), whereas control and K5 displayed comparable values. At mid‐term evaluation, a pronounced upregulation was observed in the K3 group relative to all other treatments (*p* < 0.05). Although the K5 group also showed increased expression compared to control and K1, this elevation remained lower than that observed in K3. By the end of the experimental period, maximal IL‐8 expression was detected in the K5 group, which differed significantly from the other treatments (*p* < 0.05). The K1 group also demonstrated higher expression relative to the control and K3 groups at this stage. Figure 6 shows the time‐dependent expression of *IL‐8* in kidney tissues.

**FIGURE 7:**
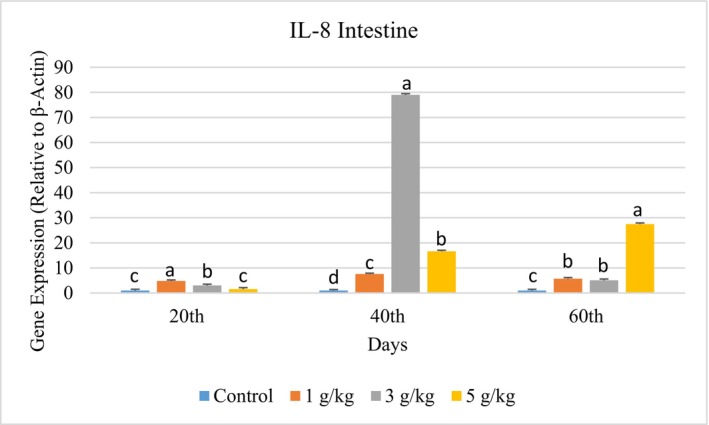
Relative IL‐8 gene expression in intestinal tissue of rainbow trout fed diets supplemented with *Hypericum perforatum* oil for 60 days. Expression levels are presented as fold change (2^−^ΔΔCt). Different letters indicate significant differences (*p* < 0.05).

Intestinal *IL‐8* expression followed a pattern largely consistent with that observed in kidney tissue. A significant elevation was detected in the K1 group at the early sampling stage, in the K3 group at mid‐term, and in the K5 group at the final sampling point (*p* < 0.05), indicating a dose‐ and duration‐dependent activation profile. Figure 7 shows the time‐dependent expression of *IL‐8* in intestinal tissues.

##### 3.2.4.3 IFN‐1

**FIGURE 8:**
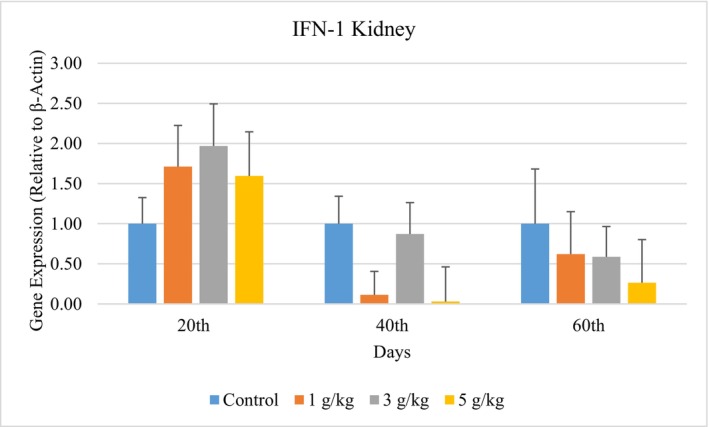
Relative IFN‐1 gene expression in kidney tissue of rainbow trout fed diets supplemented with *Hypericum perforatum* oil for 60 days. Expression levels are presented as fold change (2^−^ΔΔCt). No significant differences were detected among groups (*p* > 0.05).

**FIGURE 9:**
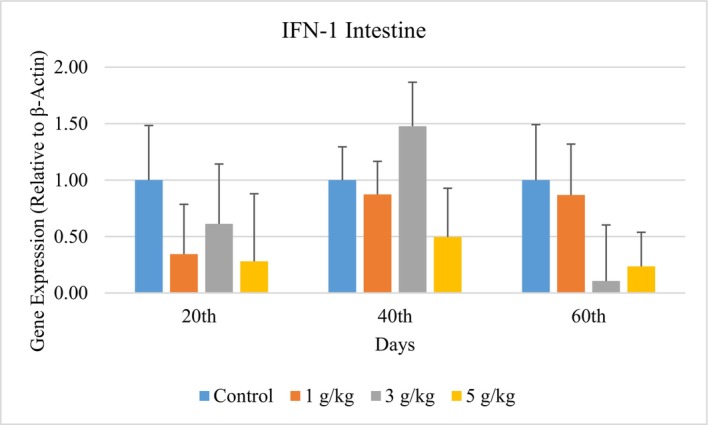
Relative IFN‐1 gene expression in intestinal tissue of rainbow trout fed diets supplemented with *Hypericum perforatum* oil for 60 days. Expression levels are presented as fold change (2^−^ΔΔCt). No significant differences were detected among groups (*p* > 0.05).

Relative *IFN‐1* gene expression levels in kidney and intestinal tissues are presented in Figures 8 and 9.

In kidney tissue, *IFN‐1* expression showed moderate fluctuations over time; however, no statistically significant differences were detected among treatment groups at any sampling point (*p* > 0.05). Although numerically higher expression levels were observed in the K3 group at the early stage and in the control group at the final sampling point, these variations were not statistically supported.

A similar pattern was observed in intestinal tissue, where *IFN‐1* expression did not differ significantly among experimental groups throughout the study period (*p* > 0.05), indicating that dietary supplementation with *H. perforatum* oil did not markedly influence interferon‐related transcriptional responses under the present conditions.

##### 3.2.4.4 TGF‐β

**FIGURE 10:**
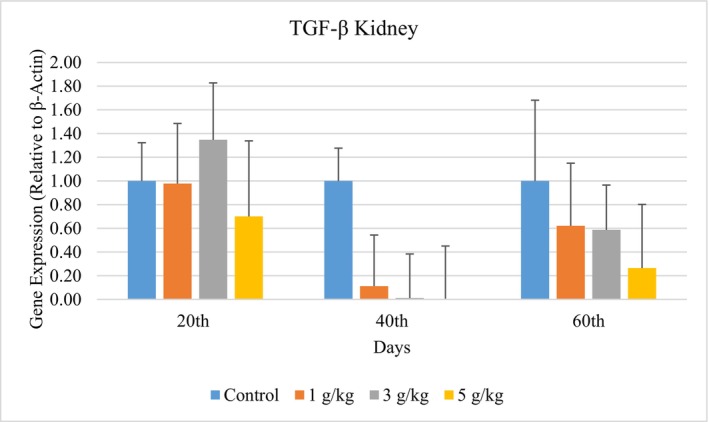
Relative TGF‐β gene expression in kidney tissue of rainbow trout fed diets supplemented with *Hypericum perforatum* oil for 60 days. Expression levels are presented as fold change (2^−^ΔΔCt). No significant differences were detected among groups (*p* > 0.05).

**FIGURE 11:**
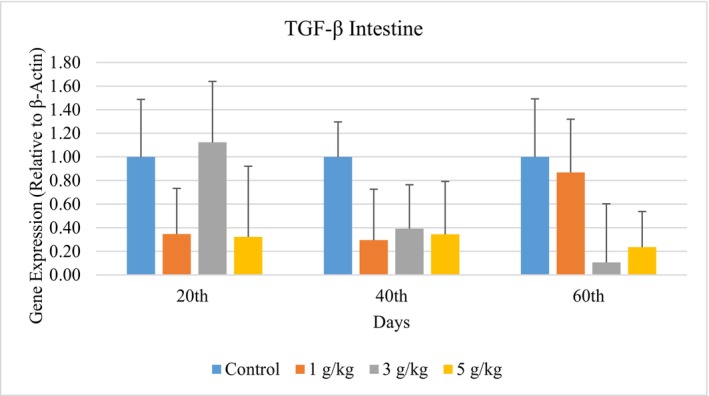
Relative TGF‐β gene expression in intestinal tissue of rainbow trout fed diets supplemented with *Hypericum perforatum* oil for 60 days. Expression levels are presented as fold change (2^−^ΔΔCt). No significant differences were detected among groups (*p* > 0.05).

Relative *TGF‐β* gene expression levels in kidney and intestinal tissues are presented in Figures 10 and 11.

In kidney tissue, *TGF‐β* expression remained relatively stable throughout the experimental period, with no statistically significant differences detected among treatment groups at any sampling point (*p* > 0.05).

A comparable pattern was observed in intestinal tissue, where TGF‐β expression did not differ significantly among groups during the study (*p* > 0.05), suggesting that dietary supplementation did not markedly alter anti‐inflammatory regulatory signalling at the transcriptional level.

##### 3.2.4.5 TNF‐α

**FIGURE 12:**
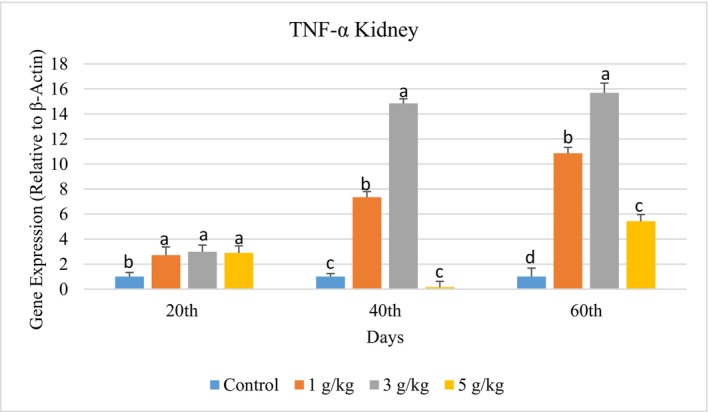
Relative TNF‐α gene expression in kidney tissue of rainbow trout fed diets supplemented with *Hypericum perforatum* oil for 60 days. Expression levels are presented as fold change (2^−^ΔΔCt). Different letters indicate significant differences (*p* < 0.05).

**FIGURE 13:**
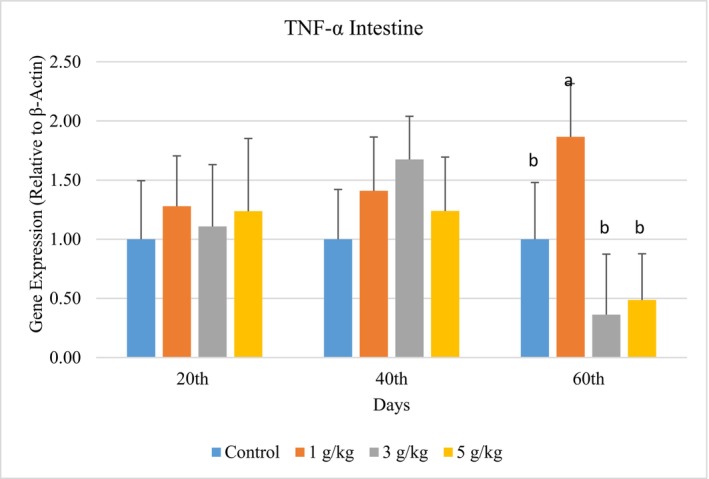
Relative TNF‐α gene expression in intestinal tissue of rainbow trout fed diets supplemented with *Hypericum perforatum* oil for 60 days. Expression levels are presented as fold change (2^−^ΔΔCt). Different letters indicate significant differences (*p* < 0.05).

Relative *TNF‐α* gene expression levels in kidney and intestinal tissues are presented in Figures 12 and 13.

In kidney tissue, *TNF‐α* expression exhibited a clear dose‐ and time‐dependent modulation. At the early sampling stage, *TNF‐α* expression was significantly elevated in all treatment groups (K1, K3, and K5) compared to the control (*p* < 0.05), indicating an early activation of pro‐inflammatory signalling. At the intermediate stage, expression levels differed significantly among all groups (*p* < 0.05), with the highest transcriptional activation observed in the K3 group, followed by K1, control, and K5. By the end of the experimental period, *TNF‐α* expression remained significantly differentiated among treatments (*p* < 0.05), with the strongest upregulation again detected in the K3 group. This sustained elevation suggests prolonged activation of inflammatory pathways at the optimal dose.

In intestinal tissue, TNF‐α expression remained comparable among groups during the early and intermediate sampling stages (*p* > 0.05). However, at the final sampling point, a significant increase was detected in the K1 group relative to the other treatments (*p* < 0.05), suggesting a delayed intestinal pro‐inflammatory response at the lower supplementation level.

##### 3.2.4.6 COX‐2

**FIGURE 14:**
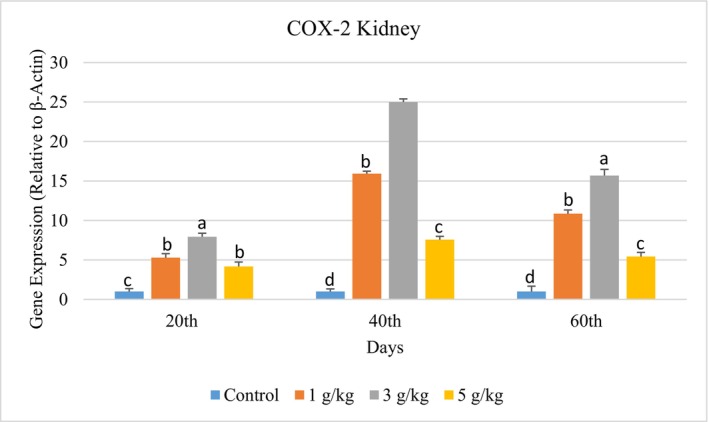
Relative COX‐2 gene expression in kidney tissue of rainbow trout fed diets supplemented with *Hypericum perforatum* oil for 60 days. Expression levels are presented as fold change (2^−^ΔΔCt). Different letters indicate significant differences (*p* < 0.05).

**FIGURE 15:**
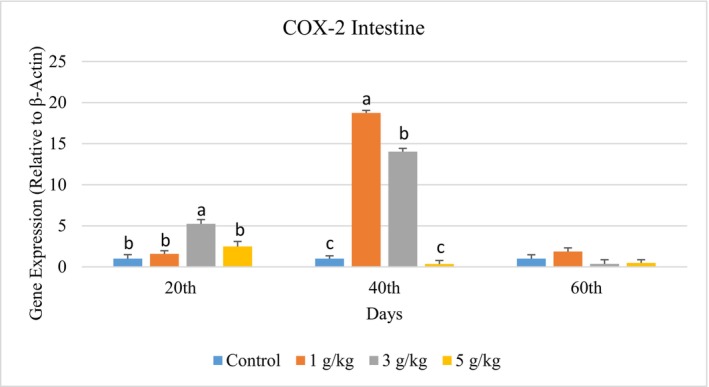
Relative COX‐2 gene expression in intestinal tissue of rainbow trout fed diets supplemented with *Hypericum perforatum* oil for 60 days. Expression levels are presented as fold change (2^−^ΔΔCt). Different letters indicate significant differences (*p* < 0.05).

Relative COX‐2 gene expression levels in kidney and intestinal tissues are presented in Figures 14 and 15.

In kidney tissue, *COX‐2* expression was significantly modulated across all sampling periods (*p* < 0.05). At each time point, the highest transcriptional activation was consistently observed in the K3 group, followed by K1, K5, and control groups, respectively. All groups differed significantly from one another (*p* < 0.05), indicating a clear dose‐dependent inflammatory response, with K3 producing the most pronounced upregulation.

In intestinal tissue, *COX‐2* expression exhibited a time‐dependent response pattern. At the early sampling stage, a significant increase was detected exclusively in the K3 group compared to the other treatments (*p* < 0.05), whereas the remaining groups did not differ significantly from each other (*p* > 0.05). At the intermediate stage, expression levels differed significantly among treatments (*p* < 0.05), with the highest values observed in K1 and K3, followed by control and K5. By the end of the experimental period, no statistically significant differences were detected among groups (*p* > 0.05), suggesting normalisation of intestinal inflammatory signalling over time.

##### 3.2.4.7 MHC‐II

**FIGURE 16:**
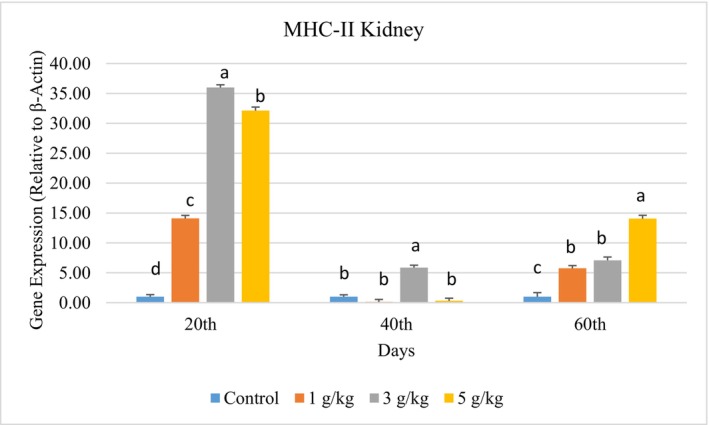
Relative MHC‐II gene expression in kidney tissue of rainbow trout fed diets supplemented with *Hypericum perforatum* oil for 60 days. Expression levels are presented as fold change (2^−^ΔΔCt). Different letters indicate significant differences (*p* < 0.05).

**FIGURE 17:**
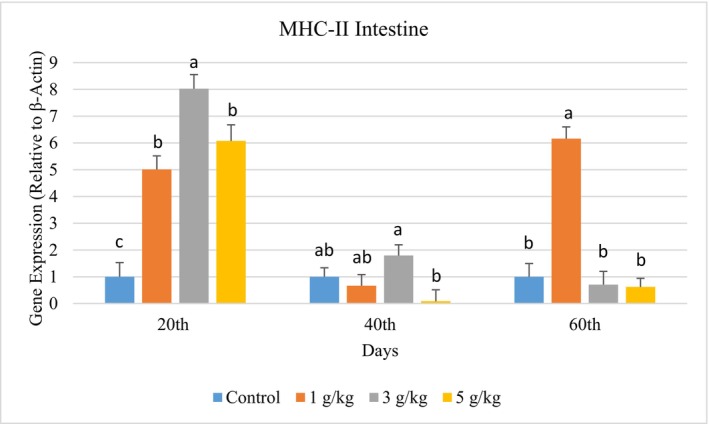
Relative MHC‐II gene expression in intestinal tissue of rainbow trout fed diets supplemented with *Hypericum perforatum* oil for 60 days. Expression levels are presented as fold change (2^−^ΔΔCt). Different letters indicate significant differences (*p* < 0.05).

Relative MHC‐II gene expression levels in kidney and intestinal tissues are presented in Figures 16 and 17.

In kidney tissue, *MHC‐II* expression was significantly influenced by dietary supplementation throughout the experimental period (*p* < 0.05). At the early sampling stage, the highest expression level was observed in the K3 group, whereas the control group exhibited the lowest value. The K5 and K1 groups showed intermediate expression levels, and all groups differed significantly from one another (*p* < 0.05). At the intermediate sampling point, *MHC‐II* transcription was significantly elevated in the K3 group compared with all other treatments (*p* < 0.05), indicating sustained activation of antigen presentation pathways at this inclusion level. At the final sampling stage, the highest expression was detected in the K5 group (*p* < 0.05). The K3 and K1 groups displayed comparable intermediate expression levels, while the control group remained significantly lower than the supplemented groups. These findings suggest a time‐dependent modulation of adaptive immune‐related gene expression.

In intestinal tissue, MHC‐II expression also varied significantly among treatments (*p* < 0.05). At the early sampling stage, the K3 group exhibited the highest expression, while the control group showed the lowest level, with significant differences among groups (*p* < 0.05). At the intermediate stage, MHC‐II expression remained significantly elevated in the K3 group compared with the other treatments (*p* < 0.05), suggesting enhanced local antigen‐presenting activity. By the end of the study, the K1 group demonstrated the highest expression level (*p* < 0.05), whereas the remaining groups showed comparatively lower values. This pattern indicates a temporally dynamic but sustained immunomodulatory effect of dietary supplementation.

#### 3.2.5 Survival Rate

**FIGURE 18:**
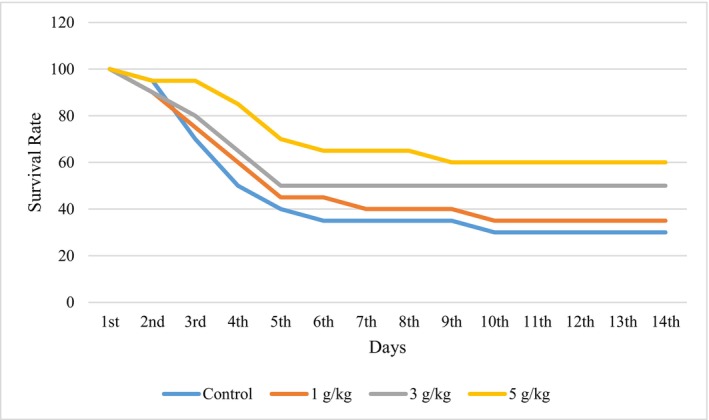
Cumulative survival rate (%) of rainbow trout following intraperitoneal challenge with *Lactococcus garvieae* . Different letters indicate significant differences among groups (*p* < 0.05).

At the end of the feeding trial, fish were challenged intraperitoneally with *Lactococcus garvieae* (1 × 10^8^ CFU per 100 μL), corresponding to the previously determined LD_50_ dose. Cumulative survival rates are presented in Figure 18.

Dietary supplementation with *Hypericum perforatum* oil influenced resistance against *L. garvieae* infection. The highest survival rate was observed in the K5 group (60%), followed by the K3 group (50%) and the K1 group (35%). The control group exhibited the lowest survival rate (30%). These findings indicate that dietary inclusion of St. John's Wort oil enhanced host resistance against bacterial infection in a dose‐dependent manner, with the strongest protective effect observed at the highest supplementation level.

## 4 Discussion

Many alternative raw materials have been tested to replace fish meal and fish oil in rainbow trout feeds. As a result of these studies, the alternative raw materials used reduced growth compared to the control feeds whose main raw materials were fish meal and fish oil (Gatlin III et al. 2007; Lim et al. 2008). Similarly, some plant raw materials added to the feed of farmed fish species have negatively affected the immune systems of fish due to the anti‐nutrient factors they contain in their structures (Montero et al. 2003, 2010; Dong et al. 2000). Some growth performance studies have indicated that feed components affect immunity and gene expression levels in fish (Spielbauer and Stahl 2005).

According to the study data, a significant increase was observed in the growth performance of rainbow trout fed with K3, especially in data such as weight gain and specific growth rate, and in addition, a decrease in FCR was detected, although not statistically significant. While K1 showed better performance in oxidative radical production on the 60th day, K3 showed the highest increase in lysozyme activities, but no general change was observed at the end of the study. MPO activity caused an increase in the K3 group on the 40th and 60th days, but this increase was not found to be statistically significant. The K3 group, which showed a particularly more significant increase in gene responses, showed a significant increase in cytokine gene expression in both kidney and intestinal tissues. In control tests against *Lactococcus garvieae* infection, the K5 and K3 groups had the best survival rate, followed by the K1 and control groups. At the end of the study, the growth performance of fish fed with the K3 group was found to be higher compared to the other groups, and the FCR increase was not found to be significantly different. Similar to our study, Sönmez et al. (2015) found an increase in the growth performance of rainbow trout fed with sage and thyme oil. However, a decrease in growth performance was found in rainbow trout fed with mint oil.

Oxidative radicals (OH‐, O2‐) are the product of the defence mechanism generated by phagocytic activity by immune cells, especially neutrophils (Anderson et al. 1992). In the study, an increase was observed in the K1 group on the 60th day of sampling, while on the 20th day, only the control group showed a lesser increase among the groups, but no significant difference was observed between the other groups. Although K1 increased more on the 40th day, this difference was not found to be significant and did not differ between the groups. Similarly, Pérez‐Sánchez et al. (2015) found that oxidative radical releases did not change in sea bream fed with the commercial product Next Enhance 150, an essential oil derivative.

The main function of lysozyme in the immune response is to break down the cell walls of bacteria and prevent them from forming colonies and aggregating (Alexander and Ingram 1992). Lysosoyme activity did not show any difference in the groups in rainbow trout fed with different amounts of St. John's Wort oil for 60 days. In this context, it can be thought that the fatty acids in St. John's Wort oil suppress the secretion of lysozyme in the body. Similar to our study, Sun et al. (2011) did not observe any change in lysozyme activity in black carp ( *Mylopharyngodon piceus* ) fed rapeseed oil. Unlike our study, an increase in lysozyme activities was observed in tilapia fed with *Citrus sinensis* (Acar et al. 2015).

Myeloperoxidase is an enzyme that stimulates macrophages and neutrophils (Grattendick et al. 2002). In our study, MPO activity increased in the K3 group, especially on the 40th and 60th days, and although this showed that neutrophils and macrophages in the K3 group were stimulated, this increase did not differ significantly from the other groups. Differently, Awad et al. (2013), fish were divided into 7 groups before being fed for 14 days with 0.1%, 0.5%, and 1% of Quercetin, 1%, 2%, and 3% of *Nigella sativa* oil and with unsupplemented commercial diet as the control. In their study, they found a significant increase in MPO activities in rainbow trout fed with black cumin oil.

IL‐1β is one of the most important and most studied cytokines, and plays a regulatory role in the defence system and triggers the release of other cytokines (Low et al. 2003). In the second and third sampling periods of this study, IL‐1β was observed to increase significantly in both kidney and intestine in the K3 group compared to the other groups. St. John's Wort oil was observed to have pro‐inflammatory properties in kidney and intestine cells of rainbow trout. Montero et al. (2010), 5 iso‐nitrogenous and iso‐lipidic diets (45% crude protein, 22% crude lipid content) were formulated. Anchovy oil was the only lipid source used in the control diet (FO), but in the other diets, fish oil was totally (100%) or partially (70%) substituted by linseed (rich in n‐3 fatty acids) or soybean (rich in n‐6 fatty acids) (100L, 100S, 70L, 70S). Fish were fed experimental diets during 80 days. Similar to our study, they found that gilthead sea bream fed with flaxseed oil increased IL‐1β gene expression.

IL‐8 is also called CXCL‐8 and, as a chemokine, increases the activity of neutrophils, T lymphocytes, and basophils. It basically acts as a proinflammatory cytokine (van der Aa et al. 2010). In a study by Pérez‐Sánchez et al. (2015), sea bream juveniles were given different doses (0, 50, 100, 200, 300 ppm) of NEXT ENHANCE150 (NE) for 9 weeks. There were no differences in the growth rates. However, their studies observed a decrease in IL‐8 activity in sea bream. On the contrary, in our study, an increase in IL‐8 gene expression was observed in both kidney and intestine in groups K1, K3, and K5, respectively, on the 20th, 40th, and 60th days of my study.

IFNs can activate inflammatory cells and are central to host defence against intracellular and extracellular pathogens (Tzianabos and Wetzler 2004). In their study, Hu et al. (2021) designed and synthesised a novel coumarin derivative, 7‐((4‐aminophenyl)diazenyl)‐4‐methyl‐coumarin (AMC). They showed that AMC can activate IFN‐related expressions. Huang et al. (2022) investigated in vitro and in vivo whether quercetin has indirect antiviral activity against Singapore grouper iridovirus (SGIV) and also examined the potential mechanism. Similarly, as a result of their study, quercetin pretreatment could induce the expression of genes involved in the type I interferon (IFN) system. In contrast, our study determined that IFN‐1 cytokines did not show a significant increase in the kidneys or intestines between groups during any of the sampling periods.

TGF‐β is a pleiotropic cytokine that regulates the development, proliferation, differentiation, migration and survival of a variety of leukocyte‐derived cells, including lymphocytes, dendritic cells, natural killer cells (NK), macrophages and granulocytes (Li and Flavell 2008; Li et al. 2006). In their study, Salem et al. (2023) describe the effects of flaxseed ( *Linum usitatissimum* ) oil (FSO) as a feed additive on growth performance, oxidative stress, immunity, and disease resistance in rainbow trout. Fish were fed different doses of FSO (0.5%, 1%, and 1.5%) twice daily for 9 weeks. Kidney TGF‐β gene expression levels did not differ between groups at week 3. However, a significant increase was observed in all groups receiving FSO supplementation at weeks 6 and 9 (*p* < 0.05), but a significant increase was observed in the FSO 0.5% group at week 6 (*p* > 0.05). Spleen TGF‐β gene expression levels were not affected by FSO supplementation at week 6. At week 3, an increase was observed in the FSO 0.5 group, while a decrease was seen in the FSO 1 and 1.5 groups (*p* < 0.05). However, at week 9, a decrease was observed in the FSO 1 group, while an increase was observed in the FSO 1.5 group (*p* < 0.05). Similarly, intestinal TGF‐β gene expression was unaffected by supplementation at week 6 (*p* > 0.05), but increased in the FSO 1.5 group at week 3 and in all experimental groups at week 9 (*p* < 0.05). In contrast to that study, our experiment determined that there was no significant increase or decrease in TGF‐β in the kidneys or intestines between the groups at any point during the sampling periods. TNF‐α is a reliable marker for determining the innate immune system and tissue damage (Secombes et al. 2001; Cho et al. 2001). In our study, an increase in the expression level of the TNF‐α gene was observed especially in the kidneys in the K3 group on the 20th and 40th days. In the intestines, a significant increase was observed in the K1 group in the 60th day sampling. A similar result of increasing the expression level of the TNF‐α gene was also found when 43% soybean meal was used in rainbow trout diets in a study conducted by Sealey et al. (2009).

Cyclooxygenase COX‐2 is an important proinflammatory cytokine (Secombes et al. 2001). In their study, Güroy et al. (2022) determined the adaptive and innate immune responses of European sea bass ( *Dicentrarchus labrax* ) to Spirulina (*Arthrospira platensis*) meal on the 30th day of the study. In the study, fish (5.74 ± 0.02 g) were fed control (C) and three different levels (1%, 2.5% and 5%) of Spirulina meal (SP1, SP2.5 and SP5) for 60 days. They observed an increase in COX‐2 in the intestines of fish fed with Spirulina diets on the 30th day of the study. In the kidneys, they determined high activity after the 60th day. In our study, very similarly, COX‐2 gene expression in the kidneys of the K3 group increased significantly in each sampling period. In the intestines, a significant increase was observed on the 20th day in the K3 group and on the 40th day in the K1 group, while no significant difference was found between the groups on the 60th day. Therefore, it can be said that St. John's wort oil stimulates leukocytes.

MHC class II molecules play important roles in cellular and humoral immune responses, various autoimmune disorders, and tissue rejection. Given these essential functions of molecules in the MHC class II group, defects in their expression appear to have serious immunopathological consequences. Failure to express these molecules results in an immune system that cannot adequately respond to foreign antigens (Klein et al. 1993). In their study, Bilen and Elbeshti (2019) administered four different concentrations of tetra ( *Cotinus coggygria* ) extract (0 [control], 4, 8, and 12 mg/100 μL) and two different positive control groups (florfenicol and doxycycline antibiotics) orally to rainbow trout twice daily using feeding needles. Blood and tissue samples were collected from the fish on days 0, 3, 7, and 10, and changes in humoral immune responses, haematology, and immunity‐related gene expression were determined. On day 3 of the study, they noted a significant increase in MHC‐II gene expression in fish treated with 4 and 8 mg tetra. Specifically, they achieved excessive upregulation in the group treated with 8 mg tetra. Similarly, on the 20th and 40th days of our study, MHC‐II increased significantly in the K3 group in the kidneys and intestines compared to the other groups. On the 60th day, an increase was observed in the K5 group in the kidneys and in the intestinal tissues in the K1 group.

When the survival rates were evaluated, the highest survival rate was seen in the K5 group, while the lowest survival rates were seen in the K3, K1, and control groups, respectively. Similar to our study, an increase in survival rates against *Vibrio anguillarum* disease was detected in sea bass fed with carvacrol (Volpatti et al. 2013). Bilen et al. (2019) created six different experimental groups to determine the therapeutic effects of aqueous methanolic extracts of beard lichen (*Usnea barbata*) against *Lactococcus garvieae* in rainbow trout and their effects on survival rate [0 mg/100 μL (Control), 4 mg/100 μL, 8 mg/100 μL, 12 mg/100 μL, 6 mg/100 μL florfenicol (positive control), 6 mg/100 μL erythromycin (positive control)]. The survival rates obtained in the group treated with 4 mg beard lichen, the group treated with 8 mg beard lichen, and the group treated with erythromycin were 73.08%, 65.38%, and 80.77%, respectively. Their results showed that the methanolic extract of beard lichen could be an effective therapeutic agent against *L. garvieae* infection in rainbow trout at a dose of 4 mg/17.41 ± 0.3 g body weight/day. In contrast to our study, the addition of the lowest dose of plant extract positively affected the survival rate. Based on this, it can be said that St. John's wort oil is more effective at higher doses and has limited side effects.

As a result, significant effects on immune responses of rainbow trout fed with St. John's Wort oil, especially on some gene expressions, were observed. Particularly, the order in gene presentations obtained as a result of the 40 and 60‐day application of the study in both the kidneys and intestines of the K3 group is remarkable. In this context, it was concluded that 40‐day K3 application was a usable method in fish production with the generally increased growth performance of the fish.

## 5 Conclusion

This study demonstrated that dietary supplementation with St. John's Wort oil positively influences growth performance, immune responses, and disease resistance in rainbow trout. Among the tested doses, the K3 (3 g/kg) application exhibited the most balanced and effective outcomes, particularly by enhancing growth parameters and significantly upregulating key immune‐related genes in kidney and intestinal tissues, especially after 40 days of feeding.

The molecular findings revealed that the K3 dose induced notable activation of pro‐inflammatory and immune‐associated genes, including IL‐1β, IL‐8, TNF‐α, COX‐2, and MHC‐II, indicating a strong immunostimulatory effect at both systemic and mucosal levels. In parallel, growth performance indicators such as final body weight, specific growth rate, and weight gain were significantly improved in this group without negatively affecting feed efficiency.

Furthermore, the challenge test with *Lactococcus garvieae* confirmed that dietary inclusion of St. John's Wort oil enhances resistance against bacterial infection, with higher survival rates observed in the supplemented groups compared to the control. Although the highest survival rate was recorded in the K5 group, the K3 dose provided a more favorable balance between immune stimulation, growth performance, and overall physiological responses.

Overall, the results suggest that dietary supplementation with St. John's Wort oil at a dose of 3 g/kg for a 40‐day period represents an effective and sustainable immunostimulant strategy in rainbow trout aquaculture. This approach offers a promising natural alternative to antibiotics by improving fish health and performance while supporting environmentally friendly and sustainable aquaculture practices.

## Author Contributions

**Soner Bilen:** study design, conceptualisation, data curation. **Mustafa Karga:** data collection, visualisation, writing – original draft. **Osman Nezih Kenanoğlu:** data collection, visualisation, writing – original draft. **Ertuğrul Terzi:** data collection, data curation.

## Funding

This work was supported by Kastamonu Üniversitesi (Grant KÜ‐BAP01/2020‐4).

## Ethics Statement

All experimental procedures were approved by the Kastamonu University Animal Experiments Local Ethics Committee (Approval No: 05, 04 February 2020).

## Conflicts of Interest

The authors declare no conflicts of interest.

## Data Availability

The authors declare that data can be provided by the corresponding author upon reasonable request.
